# The impact of a symptomics-based transitional care program on mental health and quality of life in perimenopausal women after total hysterectomy: A retrospective study

**DOI:** 10.12669/pjms.42.8.16152

**Published:** 2026-08

**Authors:** Junyu Ni, Bijun Mao, Chunyun Jin

**Affiliations:** 1 Junyu Ni, Department of Nursing, Department of Nursing, Huzhou Maternity & Child Health Care Hospital, Huzhou, Zhejiang Province 313000, P.R. China; 2 Bijun Mao, Department of Nursing, Department of Nursing, Huzhou Maternity & Child Health Care Hospital, Huzhou, Zhejiang Province 313000, P.R. China; 3 Chunyun Jin, Department of Nursing, Department of Nursing, Huzhou Maternity & Child Health Care Hospital, Huzhou, Zhejiang Province 313000, P.R. China

**Keywords:** Mental health, Perimenopausal women, Quality of life, Symptomics, Transitional care, Total hysterectomy

## Abstract

**Objective:**

To explore the impact of a symptomics-based transitional care program on mental health and quality of life in perimenopausal women after total hysterectomy.

**Methodology:**

This retrospective study was conducted using medical records from 131 perimenopausal women who underwent total hysterectomy at the Huzhou Maternal and Child Health Hospital between April 2024 and August 2025. The participants were divided into an observation group (n=67) receiving the symptomics-based transitional care program and a control group (n=64) receiving the conventional care program, based on the postoperative nursing regimen. The two groups were compared in terms of scores on the modified Kupperman Index, the Self-Rating Anxiety Scale (SAS), the Self-Rating Depression Scale (SDS), the Pittsburgh Sleep Quality Index (PSQI), the Pelvic Floor Distress Inventory-20 (PFDI-20), and the Female Sexual Function Index (FSFI). Nursing satisfaction was assessed in both groups.

**Results:**

At six months after intervention, the scores of the modified Kupperman Index, SAS, SDS, PSQI and PFDI-20 in the observation group were significantly lower, while the FSFI score was significantly higher than that in the control group (P<0.05). There was no significant difference in the nursing satisfaction between the two groups (p>0.05).

**Conclusion:**

The symptomics-based transitional care program is associated with improved postoperative mental health and quality of life in perimenopausal women undergoing total hysterectomy.

## INTRODUCTION

Perimenopause is a transitional period of reproductive aging, characterized by fluctuating hormone levels. It has been reported that there are approximately 130 million perimenopausal women in China, and this number is projected to reach 280 million by 2030.^1^,^2^ Approximately 80% of women have suffered from at least one menopause-related symptom, including hot flashes and sweating, depressed mood, sleep disorders, memory loss, dyspareunia, vaginal dryness, urinary incontinence, paresthesia, and somatic pain, which severely impair the quality of life and mental health of patients.^1^–^3^ Furthermore, perimenopausal women have a higher incidence of common gynecological diseases, such as uterine leiomyoma and adenomyosis, which for women without fertility requirements are routinely treated by hysterectomy.^4^

Studies have indicated that age-standardized prevalence of hysterectomy in urban China was 2.36% (provincial range: 0.925%-3.26%), with perimenopausal women aged 55-59 (7.61%) having the highest prevalence.^5^,^6^ However, total hysterectomy increases the risk of complications such as pelvic floor dysfunction, stress urinary incontinence, and lower extremity deep vein thrombosis, and has substantial psychological and physiological implications for the patients.^6^,^7^ Therefore, it is crucial to develop nursing management strategies and effective intervention measures targeting the postoperative symptoms of perimenopausal women who have undergone total hysterectomy.

A concept of symptomics was first proposed by Fried et al.^8^ in 2015. This approach moves beyond category-based diagnoses by treating individual symptoms as the core and measurable components of mental disorders. Studies have demonstrated that targeted assessment and intervention focusing on core and bridge symptoms can effectively improve symptom management outcomes in patients, prompting the U.S. National Institute of Nursing Research to advocate for advancing symptom science in nursing.^8^–^10^ However, the impact of symptomics on gynecological nursing is unclear. Therefore, the study aimed to explore the impact of a symptomics-based transitional care program on mental health and quality of life in perimenopausal women after total hysterectomy.

## METHODOLOGY

This retrospective study was conducted using medical records from 131 perimenopausal women who underwent hysterectomy at the Huzhou Maternal and Child Health Hospital between April 2024 and August 2025. Based on the postoperative nursing plan, the patients were divided into an observation group (symptomics-based transitional care) and a control group (conventional nursing care).

### Ethical approval:

The ethics committee of our hospital approved this study with the number: 2024-R-019; Date: December 26, 2024.

### Inclusion criteria:

- Age ranged from 40 to 60 years.
- ≤12 menstrual cycles within 12 months before surgery.
- Ovaries preserved after hysterectomy.
- Diagnosis of benign gynecological disease.
- Patients whose baseline sexual function, as assessed by the Female Sexual Function Index (FSFI), was within the normal range.
- Follow-up duration ≥6 months.
- Complete clinical data available.

### Exclusion criteria:

- Presence of malignant tumors.
- Vulvovaginal injury or genital tract malformations.
- History of pelvic surgery, diabetes, heart disease, chronic respiratory disease, stroke, epilepsy, other neurological disorders, or psychiatric disorders (including depression).
- Concomitant vaginal or urinary tract infections.

### Nursing protocols:

All staff received standardized training.

### Control group:

Patients received conventional nursing care. During hospitalization, nurses communicated with patients to familiarize them with the hospital environment, department layout, and medical staff. Medical records were established, and patients were provided with explanations regarding their disease condition and surgical treatment, along with relevant educational materials. Psychological support was offered through patient counseling and encouragement to alleviate anxiety and negative emotions.

Perioperative nursing included guidance on daily living, diet, and rest to promote healthy lifestyle habits. Postoperatively, nurses closely monitored patients’ clinical status and vital signs, provided routine wound care, and guided rehabilitation exercises, medication use, and the prevention and management of potential complications.

Before discharge, nurses conducted discharge education through face-to-face communication, assessed patients’ self-care abilities—particularly regarding catheter maintenance when applicable—and reinforced relevant guidance. Follow-up management was conducted using a standardized follow-up template, which included maintaining patient contact information and scheduling regular postoperative reviews, including telephone follow-up at two weeks and outpatient visits at one, three and six months postoperatively. During follow-up, patients’ recovery status and self-care practices were assessed each time; patients’ questions were addressed, and further guidance according to the inquiries was provided as needed.

### Observation group:

Patients received the symptomics-based transitional care in addition to the routine nursing. The details of the approach are summarized in Table-I.

**Table-I:** Symptomics-based transitional care program.

Intervention Focus	Specific Content	Implementer	Timing & Frequency	Location & Method
Management of Postoperative Symptoms	Incision pain: Keep wound dry to prevent infection and have moderate activity. Abdominal distension and constipation: Low-salt high-fiber diet plus sufficient drinking water to prevent constipation. Slight vaginal discharge and frequent micturition: Void regularly and avoid urinary retention. Fatigue and low-grade fever: Stick to regular daily routine, supplement calcium and vitamin D; apply hormone therapy as physician ordered for severe symptoms and ease mood disturbance.	Nurses	/	Ward: Face-to-face communication between nurse and patient Clinic: Face-to-face consultation with doctor for medication guidance
Integrated Chinese-Western medication therapy to improve menopausal symptoms	During hospitalization: Face-to-face communication was conducted with patients to explain study details, patients were informed of the care program, and add patients/main caregivers to a WeChat group; Gynecologists were added to the WeChat group to introduce doctors and clinic hours to patients and their families; When patients develop menopausal symptoms, they are scheduled for regular clinic follow-ups and medication guidance.	- Responsible nurse - Gynecologist - Traditional Chinese Medicine physician	Monthly, starting from the onset of symptoms	Ward: Face-to-face communication between nurse and patient Clinic: Face-to-face consultation with doctor for medication guidance
Strengthen pelvic floor function exercise to accelerate recovery of pelvic floor & sexual function and enhance patient confidence	Patients and families were instructed to perform transversus abdominis-pelvic floor muscle co-contraction training: The bladder was emptied before training; patients sit on a Bobath ball in an unsupported sitting position, keep the trunk stable; patients were instructed to exhale while contracting the lower abdominal wall (close to the spine to maximize transversus abdominis contraction), then contract vaginal and perianal muscles as much as possible, lift the vagina/anus, hold for 3–5 seconds, relax for 10 seconds, and repeat for 15 minutes. Evaluation standard: The examiner places one hand on the patient’s anus and the other on the abdomen; coordinated contraction of perianal and abdominal muscles indicates effective exercise. Training requirements: 3 sessions/week in the first 2 weeks; ≥2 sessions/week for the next 3 months; ≥1 session/week after 3 months, continuing for 6 months. WeChat video check-ins and family supervision are required each time.	Nurses	3 sessions/week in the first 2 weeks; ≥2 sessions/week for the next 3 months; ≥1 session/week after 3 months, continuing for 6 months	Home exercise for patients; supervision via WeChat/video
Nurse-patient communication (Q&A) to identify problems promptly, help patients establish cognition, and build health beliefs	Discharge education and guidance manual distribution on the day of discharge; ii. Questions in the WeChat group were collected at 6 PM daily within 1 month after discharge, answer questions on Tuesdays/Thursdays/ Saturdays weekly; patients with severe issues were contacted via WeChat video calls or home visits, and were also scheduled for specialist clinics; Patients are contacted twice weekly (including science popularization, psychological counseling, follow-up reminders) within 2–3 months after surgery; once weekly within 3–6 months after surgery.	WeChat group dedicated nurse	Twice weekly within 2–3 months after surgery; once weekly within 3–6 months after surgery	WeChat/video; home visits (if needed)

### Observation indexes:

- Perimenopausal symptoms were evaluated using the modified Kupperman rating scale, which includes 13 items such as insomnia, irritability, hot flashes, sweating, and dyspareunia. Each item is scored on a 4-point scale (0–3) based on symptom severity and then multiplied by its corresponding weighting coefficient. The weighted scores of all items were summed to obtain a total score, with lower scores indicating milder perimenopausal symptoms.
- Anxiety and depression were assessed using Zung’s Self-Rating Anxiety Scale (SAS) and Self-Rating Depression Scale (SDS). Both scales consist of 20 items scored on a 4-point Likert scale (1–4). Higher total scores indicate greater severity of anxiety or depressive symptoms.
- Sleep quality was assessed using the Pittsburgh Sleep Quality Index (PSQI). The PSQI comprises 19 self-rated items and five observer-rated items; only the 18 scored self-rated items were included in the analysis. The seven component scores are each scored from 0 to 3 and summed to yield a total score ranging from 0 to 21. A PSQI score ≥7 was considered indicative of a sleep disorder, with higher scores reflecting poorer sleep quality. Participants completed the questionnaire within approximately 5–10 minutes.
- Pelvic floor function was evaluated using the Pelvic Floor Distress Inventory-20 (PFDI-20), which includes 20 items across three domains: bladder symptoms, bowel symptoms, and pelvic organ prolapse symptoms. Each item was scored on a 5-point scale (0–4), and domain scores were standardized to a percentage scale. Higher scores indicate more severe pelvic floor dysfunction.
- Sexual function was assessed using the Female Sexual Function Index (FSFI). The FSFI contains 19 items across six domains: sexual desire, arousal, lubrication, orgasm, satisfaction, and pain. The total score ranges from 0 to 36, with higher scores indicating better sexual function and quality of sexual life.
- Nursing satisfaction was evaluated using a self-developed nursing satisfaction questionnaire covering health education, service timeliness, professional skills, management quality, communication, and follow-up. The questionnaire included 20 items rated on a 5-point Likert scale (1 = “very dissatisfied” to 5 = “very satisfied”), with a total score of 100 points. Scores of 80–100 indicated “very satisfied”, 60–79 indicated “generally satisfied”, and <60 indicated “dissatisfied”. Overall nursing satisfaction was calculated as: (number of “very satisfied” cases + number of “generally satisfied” cases) / total number of cases × 100%, with higher values indicating greater nursing satisfaction.
- All indexes were collected at six months after intervention. The primary outcomes were the scores of the modified Kupperman rating scale, the scores of the Zung’s SAS and SDS. The secondary outcomes were the scores of the PSQI, PFDI-20, and FSFI.

### Statistical analysis:

SPSS/pc statistical software (version 25.0; IBM Corp, Armonk, NY, USA) for Windows was used. The count data were expressed in the form of n (%), and the differences between groups were tested by Chi-square test or Fisher’s exact test. Visual (histogram and probability map) and analytical (Kolmogorov-Smirnov/Shapiro-Wilk test) methods were used to assess whether the variables follow a normal distribution. Measurement data that follow a normal distribution were expressed as mean ± standard deviation (SD). The difference between the two groups was tested using an independent-samples t-test. The paired t-test was used for within-group comparison. Data with a non-normal distribution were reported as median and interquartile range (IQR). For non-normally distributed continuous variables, the Mann-Whitney U test was used for between-group comparisons, and the Wilcoxon signed-rank test was used for within-group comparisons of paired pre- and post-intervention outcomes. Statistical significance was set as p<0.05.

## RESULTS

This retrospective study included data from 131 patients aged 40-60 years, with a median age of 48 (46-51) years. There were 67 cases in the observation group and 64 cases in the control group. There was no significant difference in age, BMI, education level, monthly household income, marital status, and operation time between the two groups (p>0.05; Table-II).

**Table-II:** Comparison of baseline characteristics between the two groups.

Variables	Observation group (n=67)	Control group (n=64)	Z/t/χ^2^	P
Age (year), median (IQR)	49 (46-51)	48 (46-52)	-0.058^a^	0.954
BMI (kg/m²), mean±SD	24.0±2.5	23.8±2.9	0.540^b^	0.590
Education level, n(%)			1.433^c^	0.489
Junior high school and below	24 (35.8)	21 (32.8)		
High school	31 (46.3)	26 (40.6)		
College degree or above	12 (17.9)	17 (26.6)		
Monthly household income, n(%)			1.661^c^	0.436
<3000	21 (31.3)	17 (26.6)		
3000~5000	24 (35.8)	30 (46.9)		
>5000	22 (32.8)	17 (26.6)		
Marital status, n(%)			1.227^c^	0.268
Married	64 (95.5)	58 (90.6)		
Divorced/widowed/unmarried	3 (4.5)	6 (9.4)		
Operation time (hours), median (IQR)	3.7 (2.7-4.5)	3.6 (2.9-4.8)	-1.053^a^	0.292

***Note:*** IQR, interquartile range; SD, standard deviation.

a , Mann-Whitney U test;

b , independent-samples t-test;

c , Chi-square test.

Before intervention, there was no significant difference in Kupperman modified score, SAS, SDS, PSQI, pfdi-20, and FSFI between the two groups (p>0.05). After six months of intervention, the Kupperman modified score, SAS, SDS, PSQI and PFDI-20 scores of the two groups significantly decreased, and were markedly lower in the observation group compared to the control group. Meanwhile, post-intervention FSFI scores increased in both groups, and the observation group had a significantly higher score than the control group (p<0.05; Table-III). This result shows that after six months of intervention, the improvement of perimenopausal symptoms, negative psychology, sleep quality, pelvic floor muscle function, and sexual life quality in the observation group is better than that in the control group.

**Table-III:** Comparison of Kupperman modified score, SAS, SDS, PSQI, PFDI-20, and FSFI scores between the two groups.

Variables	Observation group (n=67)	Control group (n=64)	t/Z	P
***Before intervention***				
Kupperman modified score, median (IQR)	16 (12-19)	15 (12-20)	-0.309^a^	0.757
SAS, mean±SD	56.6±8.3	58.4±10.2	-1.094^b^	0.276
SDS, median (IQR)	53 (51-59)	54.5 (51-62.5)	-1.816^a^	0.069
PSQI, mean±SD	13.5±2.6	14.1±2.8	-1.256^b^	0.211
PFDI-20, median (IQR)	62 (54-68)	61.5 (54-68)	0.198^a^	0.843
FSFI, median (IQR)	18 (15-20)	16.5 (15-20)	-1.266^a^	0.206
***After intervention***				
Kupperman modified score, median (IQR)	13 (11-14)*	14 (13-16)*	-3.168^a^	0.002
SAS, median (IQR)	45 (41-52)*	48 (43.5-55.5)*	-2.446^a^	0.014
SDS, median (IQR)	42 (40-47)*	46 (42-52)*	-3.498^a^	<0.001
PSQI, median (IQR)	9 (8-11)*	11 (9-12)*	-4.668^a^	<0.001
PFDI-20, median (IQR)	39 (38-42)*	47.5 (43.5-52)*	-5.925^a^	<0.001
FSFI, mean±SD	25.4±3.6*	23.4±4.2*	2.900^b^	0.004

***Note:*** SAS, self-rating Anxiety Scale; SDS, self-rating Depression Scale; PSQI, Pittsburgh Sleep Quality Index; PFDI-20, Pelvic Floor Distress Inventory-20; FSFI, Female Sexual Function Index; IQR, interquartile range; SD, standard deviation.

a , Mann-Whitney U test;

b , independent-samples t-test;

* , P<0.05 for comparison within group before and after intervention.

After six months of nursing intervention, 42 (62.7%) patients in the observation group were very satisfied with the nursing care, 23 (34.3%) were generally satisfied, and two (3.0%) were not satisfied. In the control group, 32 (50.0%) patients were very satisfied, 29 (45.3%) were generally satisfied, and three (4.7%) were not satisfied. The overall satisfaction rates of both groups were comparable (p>0.05; Table-IV).

**Table-IV:** Comparison of nursing satisfaction between the two groups.

Group	Very satisfied	Generally satisfied	Dissatisfied	Overall Satisfaction
Observation group (n=67)	42 (62.7)	23 (34.3)	2 (3.0)	65 (97.0)
Control group (n=64)	32 (50.0)	29 (45.3)	3 (4.7)	61 (95.3)
*P*				0.675*

***Note:***

* , Fisher’s exact test.

## DISCUSSION

This study investigated the impact of a symptomics-based transitional care program on mental health and quality of life in perimenopausal women after total hysterectomy. Compared with conventional care, the symptomics-based intervention was associated with significant improvements in menopausal symptoms, psychological well-being, pelvic floor function, sexual function, while nursing satisfaction was comparable between groups. The results suggest that symptomics-based transitional care may be a feasible and effective nursing strategy for postoperative management in this population.

The significantly lower modified Kupperman Index score observed in the observation group at six months post-intervention is consistent with the research results of Wei et al.^11^ Perimenopausal women have fluctuations in hormone levels due to ovarian function decline, and total hysterectomy will further disrupt the reproductive endocrine homeostasis, exacerbating menopausal symptoms such as hot flashes, insomnia, and mood swings.^5^–^7^ However, conventional care mostly focuses on basic nursing, such as postoperative wound healing and infection prevention, and lacks targeted intervention for menopausal-specific symptoms.

The symptomics-based transitional care program incorporates preoperative symptom screening and postoperative dynamic assessment to identify related symptoms, such as hot flashes and night sweats, sleep disturbances, and emotional abnormalities, based on which personalized intervention strategies are developed and implemented. For example, for patients with hot flashes and night sweats, providing dietary guidance (such as reducing intake of spicy and irritating foods), lifestyle guidance (such as wearing breathable clothing and maintaining a regular schedule), and psychological counseling may effectively alleviate menopausal discomfort. This approach is consistent with the symptomics framework of precision identification, classified intervention, and dynamic adjustment.^11^,^12^

Patients receiving symptomics-based transitional care program had lower SAS and SDS scores after intervention, indicating that symptomics-based transitional care can effectively improve postoperative anxiety and depression in patients. This is consistent with the research results of Wang et al.^13^ For perimenopausal women, total hysterectomy is not only a physical trauma but also a psychological stress.^14^ In addition, the persistent impact of postoperative physical discomfort is likely to lead to the emergence of negative emotions such as anxiety and depression.^13^ Under the conventional nursing model, psychological intervention is primarily limited to general health education, which cannot meet patients’ individual psychological needs. In contrast, the transitional care program in this study, through measures such as regular one-on-one psychological interviews, enabled patients to cope with physical and mental changes resulting from the surgery, helped relieve negative emotions, and enhanced their psychological resilience in managing the disease.^15^ The results of this study further confirm the advantage of a nursing model that combines precision symptom intervention and psychological support in improving postoperative psychological status.

Additionally, patients receiving symptomics-based transitional care program had significantly lower PFDI-20 scores and significantly higher FSFI scores than patients receiving conventional care, indicating improved pelvic floor function and sexual function. These findings are consistent with those reported by Shi et al.^15^ The symptomics-based transitional care program integrates regular postoperative pelvic floor function assessments to identify pelvic floor dysfunction–related symptoms with sexual dysfunction symptoms, enabling the development of targeted, long-term rehabilitation strategies. For patients with mild pelvic floor dysfunction, individualized pelvic floor muscle training was implemented and dynamically adjusted based on functional recovery. For patients with concurrent dyspareunia, hormone replacement therapy and sexual health guidance were facilitated in collaboration with gynecologists. Such a comprehensive, symptom–based approach effectively improved recovery of pelvic floor and sexual function.^16^–^18^

The nursing satisfaction is comparable between the two groups (97.0% vs. 95.3%). This finding suggests that symptom-based transitional care and conventional nursing care yield comparable effects on patient nursing satisfaction.^19^ Symptom-based transitional care is an extended nursing model centered on ongoing symptom management. It differs from conventional nursing care by emphasizing post-discharge follow-up, continuous symptom monitoring, and individualized guidance. Such an approach may increase the intensity of nursing interventions and require greater time and effort from patients, potentially reducing acceptance. Nevertheless, patients in the observation group maintained a high level of satisfaction comparable to that of the control group, indicating good acceptance of symptom-based transitional care. A possible explanation is that this care model more closely aligns with patients’ actual health needs after discharge. Providing targeted symptom interventions effectively alleviates post-discharge discomfort and may reduce the risk of symptom recurrence, thereby offsetting the inconvenience associated with increased nursing procedures and enhancing willingness to actively engage in nursing care.

### Limitations:

First, this was a single-center retrospective analysis based on medical records, in which patients were grouped based on their nursing protocol rather than being randomly assigned, and propensity score matching or multivariate regression correction was not used, which may have selection bias and limit the generalizability of the findings. The small sample size and short follow-up period may reduce the study’s statistical power and limit its ability to evaluate long-term outcomes of the care program, particularly effects beyond one year. Second, the symptomics-based transitional care was performed primarily based on common postoperative symptoms, without the use of standardized symptomics assessment tools or advanced analytical methods. Third, potential influencing factors (age, educational level, comorbidities, etc.) were not systematically examined for their associations with symptom formation. Fourth, nursing satisfaction was evaluated using a self-developed questionnaire. Although pilot testing and expert review were conducted to enhance its validity and reliability, given that it is not a widely recognized assessment tool, concerns regarding its measurement validity and reliability may still persist. Fifth, the cost-effectiveness of the transitional care program was not evaluated. Economic factors such as nursing workforce input and patient medical expenditures were not analyzed, limiting the ability to inform clinical decision-making and large-scale implementation of the approach. Finally, due to the retrospective and observational nature of the study, the study can only demonstrate an association between the two nursing models, and cannot be used to infer causality.

### Recommendations:

Further multicenter, prospective studies with larger sample sizes and longer follow-up durations are needed to validate the results. Moreover, integrating standardized symptomics assessment tools and formal cost-effectiveness evaluations would provide a more robust evidence base to support the widespread clinical application of symptomics-based transitional care programs.

## CONCLUSION

The symptomics-based transitional care program is associated with improved menopausal symptoms, psychological status, pelvic floor function, and sexual function in perimenopausal women after total hysterectomy, and its nursing satisfaction is comparable to that of conventional nursing care. Despite certain limitations, this study provides new ideas and methods for nursing intervention in perimenopausal patients after hysterectomy. Additional prospective studies with larger sample sizes and longer follow-up periods should be conducted to improve the symptomics assessment system, conduct cost-effectiveness analyses, further optimize the care program, and promote the precise and standardized development of nursing care for perimenopausal patients after hysterectomy.

### Authors’ Contributions:

**JN:** Literature search, study design and manuscript writing. **BM and CJ:** Data collection, data analysis and interpretation. Critical review. **JN:** Manuscript revision and validation and is responsible for the integrity of the study. All authors have read and approved the final manuscript.
